# Observations on the Plague

**Published:** 1812-04

**Authors:** Douglas Whyte


*To the Editors of the Medical and Physical Journal.*

GENTLEMEN,

IF you think the "Remarks" which accompany this worthy of a place in your Journal, they are much at your service: they came into my hands by accident, and I have reason to believe never were made public. The wide space through which your Journal circulates, affords an easy mean of making known any thing, however trifling, which may occur to medical practitioners, and of preserving many an useful fugitive opinion, which would otherwise be lost.

There cannot exist the least doubt but that these Remarks contain the true opinion of Dr. Whyte. I am informed by the gentleman who gave them to me, a captain in the royal navy, who was on terms of intimacy with him in Egypt, that he used almost to live in the hospitals, and apartments filled with patients in all stages of the disorder on which he writes; and even went so far as to inoculate himself at different times, to shew the impossibility of producing the disease in question, either by infection or contagion. It may not be amiss, however, to state that the doctor died in Asia, but whether by plague or otherwise I cannot say.*

Having seen some papers in your Journals on the hybernation of Swallows, without attempting to contradict or confirm the opinion, I send you the following extract from a journal kept by me on board H.M.S. Raleigh, on the coast of Africa. "A little to the northward of the Canary Islands, and between these islands and the continent, on the forenoon of the 17th of March, 1809, observed a flight of swallows (species unknown) probably two hundred, steering north-north-west by compass, a few apparently much fatigued rested for some minutes on our yards and rigging, and then followed their comrades." The few opportunities

* It was reported in England, that Dr. Whyte at length became the victim of his temerity, though he resisted the infection of plague on the first inoculation.—EDITORS.

allowed allowed me of communicating with England, will, I hope, be a sufficient excuse for stating this fact out of order and time. I am, Gentlemen,

Your very obedient Servant,

*Quiberon Bay, Jan.* 20, 1812. W. S. KIDD,

Surgeon of H. M. S. Pelorus.

OBSERVATIONS *on the* PLAGUE, *by* DR. DOUGLAS WHYTE.

"A sea captain, who had been seventeen years in the Smyrna trade, informs me, that, without he or his ship's company receiving any injury, he has stowed and unstowed cottons which had been packed and carried by men, some of whom he has known to have died of the plague while they were even so employed.

"Now, as the climate of Asia Minor is so favorable to the progress and propagation of this disease, why, if so contagious as represented, did it not communicate itself to men predisposed by the nature of their employments, their manners, and peculiar mode of living, and who so freely exposed themselves to its influence? Were the disease communicable by effluvia locked up in cotton bales, it would be so communicated every day; for it is matter of public notoriety, that, by bribing the Greek bishop, from whose report bills of health are granted, every ship may at any time, when the plague is not uncommonly prevalent, sail for England with clean bills: although the cottons with which she is loaded have been packed and carried, as hinted above, *by men who have died of the plague!*

"This is a thing well known to every merchant and shipmaster, and at any of the ports, (Constantinople, I believe, alone excepted,) the required certificate may be obtained for five or six hundred piasters; that, notwithstanding this, the plague is *not* imported, although in some countries, as Holland, there is little or no quarantine: the most convincing proof is afforded that it is not of so contagious a nature as *primâ facie* we had been taught to believe. Nor is this all, such is the influence of money among the ill-paid officers of health, both in England and other countries, that the gentleman to whom I am indebted for this information assures me he has known poor men, and those who declined stooping to such arts, detained to perform a strict and tedious quarantine, although they came (with foul bills of health it is true) from a port (Smyrna) where the plague had ceased nearly a whole month previous to their departure; while rich Jewish merchants, with equally or more unclean bills from the port of Mogadore, where the plague was known at the time of their departure to rage with much inveteracy, have have by dint of money, after a few trifling forms, been suffered to proceed. This happened in England only a couple of years ago, yet no plague was thereby introduced, although, as being Jews, the hazard of introduction, were it so portable as represented, would have been very great; for that people, as well as the Turks, acting agreeably to both the spirit and letter of the law, attend the sick with the same pious care, when afflicted with plague, as when ill of any other disease.

"Prior to the recent improvement in English Lazarettoes, ships from the Levant with foul bills were obliged, previous to coming home, to call at some port in health, there land their cargoes, and allow it to remain during forty days; yet ways and means were often discovered so to manage matters, that, applying a figure in rhetoric, in the same manner as bills are read in parliament, a part has been put for the whole; a boat-load of goods has been sent ashore one hour, and re-embarked the next; they have remained the limited time, enjoying the pleasures of the place, and returned to England much pleased with their dexterity in eluding the well-meant regulation of a provident legislature. I am told too, that sometimes a few hundred piastres, properly disbursed, have procured the necessary certificates, without going over the farce of sending *any* part of the cargo on shore. The crime of overlooking the religious performance of the required quarantine, or of bribing officers of health to grant clean bills, when committed by those who believe plague to be a contagious disease, exceeds the measure of common delinquency, and for which no common punishment ought to be annexed; it is equally heinous, if not more so, than even treason itself. Were the plague as contagious as some pretend to believe, it would be impossible for you to give the sword of iniquity a wider field or a sharper edge. What! would you involve a whole nation in common ruin? Would you wilfully disseminate among your friends and countrymen what is infinitely more dreadful than fire or sword? Would you diabolically attempt to exterminate a whole people? If you lend a deaf ear to the remonstrance of humanity, are you lost to friendship? Are you lost to patriotism? Is the world to you an universal blank? Have you neither a wife, mother, sister, brother, or friend? No, sure! In him who is capable of such an act, the social instinct must be extinguished, not a principle of virtue can remain! Yes, those boxes and bales you are afraid to open or to touch, you would send, like the gift of Jupiter to Pandora, to spread sickness and desolation throughout your native land!!!

"Were it practicable to convey the plague from one country country to another, it would not surely have defied for many hundred years the numerous attempts that have been made to introduce it into England by so many of her unprincipled sons; yet, for the honor of human nature, I do not conceive that either one or the other class of these delinquents seriously believe that the plague is so contagious as they are sometimes, from interest or vanity, tempted to describe. At any rate they so invert the telescope when it suits them, that what was formerly a gigantic crime, dwindles at the touch of gold to a venial transgression.

"The Turks formerly considered, and many of them do so to this day, the healing art as a species of necromancy, and many of the practitioners who come among them accommodate themselves with becoming and *profitable* complaisance to the opinions and prejudices of their ignorant employers. I was consulted some time since by a reis or sea-captain, who eight months ago had one of his leg's fractured, between two and three inches of the tibia had exfoliated; the external wound was healed up, there was hope of the bone, or a callous from it being produced, and deformity entirely prevented; but the poor man, misled by charlatans, whom he had formerly consulted, wished me to cut open the closed wound, and introduce a portion of bone from the leg of a large dog, to supply the place of the exfoliated tibia.

"On the same day I was consulted by an Imaum, respecting a fistula in ano, of which he expected to be cured without an operation. I told him it was impossible: but, would the Mussulman have advanced a few score of piasters, a Venetian surgeon, who stood by, had the assurance to offer to undertake it; and asserted that there were many internal remedies by which it could be easily accomplished.

"There are said to be in Constantinople and its environs, at the lowest computation, ten thousand medical practitioners; and they are not uncandid who maintain that not above three or four in the thousand have the smallest pretensions to a liberal, and still less to a regular professional, education. The Jewish, Greek, or Armenian, dragoman, who visits patients along with the self-made physician to-day, sets up for himself to-morrow; and, as scientific treatment is out of the question, the servant is in every respect equal to his lord.

"In chronic complaints, a species of palliation is all that is in general attempted. I have been told many of them are so unprincipled, that they would scarcely improve their practice if they had it in their power; it would be suffering the prey to escape them which had cost some trouble to allure into their toils; and such surmises are not improbable, for, for, in this benighted country, the actual cautery continues still to be in vogue.

"Were the plague so contagious as is often represented, the whole earth would have been long before now changed into a desert. Are there any instances, I would ask, of persons living, eating, and sleeping, with, and even sucking the breasts of, small-pox patients, without receiving the disease? Is there a single instance on record where a syphilitic nurse has failed to infect the child she suckled? The same question may be repeated concerning Psora, Rubeola, and the whole catalogue of contagious diseases. Exceptions *may* exist, but they must be solitary ones, and it has not been my fortune to observe them. In plague, however, such instances are frequent; and almost every Frank with whom I am acquainted, both at Smyrna and Constantinople, has recounted to me cases, not apprehended to be plague till a little before and sometimes after the patient's death, where every person in the family had exposed himself with impunity to what was believed to be a genuine source of infection.

"Of the ten thousand soi-disant surgeons and physicians of Constantinople, if there existed one, whom either Pæon or Æscalapius would own, instead of shrinking cowardly from the enterprise, he would at least reconnoitre his enemy; if unable to cope with him in the open field, he might observe his movements from afar: distant war is not always unsuccessful; Ovid says, 'Achilles had only one mortal part, and Paris shot him through the heel!'"

